# Characterization of genetic predisposition to molecular subtypes of breast cancer in Brazilian patients

**DOI:** 10.3389/fonc.2022.976959

**Published:** 2022-08-31

**Authors:** Daniele Paixão, Giovana Tardin Torrezan, Karina Miranda Santiago, Maria Nirvana Formiga, Samuel Terkper Ahuno, Emmanuel Dias-Neto, Israel Tojal da Silva, William D. Foulkes, Paz Polak, Dirce Maria Carraro

**Affiliations:** ^1^ Oncogenetics Department, A.C.Camargo Cancer Center, São Paulo, SP, Brazil; ^2^ Clinical and Functional Genomics Group, International Research Center/CIPE, A.C.Camargo Cancer Center, São Paulo, SP, Brazil; ^3^ National Institute of Science and Technology in Oncogenomics and Therapeutic Innovation (INCITO), São Paulo, SP, Brazil; ^4^ Tri-Institutional PhD Program in Computational Biology and Medicine, Weill Cornell Medicine, New York, NY, United States; ^5^ Genomic Medicine Group, - International Research Center/CIPE, A.C.Camargo Cancer Center, São Paulo, SP, Brazil; ^6^ Bioinformatics and Computational Biology Group, - International Research Center/CIPE, A.C.Camargo Cancer Center, São Paulo, SP, Brazil; ^7^ Program in Cancer Genetics, Department of Oncology and Human Genetics, McGill University, Montreal, QC, Canada; ^8^ Computational Biology, C2i Genomics, New York, NY, United States

**Keywords:** breast cancer, hereditary cancer, multigene panel, cancer genetics, molecular subtype of breast cancer

## Abstract

**Introduction:**

*BRCA1* and *BRCA2* germline pathogenic variants (GPVs) account for most of the 5-10% of breast cancer (BC) that is attributable to inherited genetic variants. *BRCA1* GPVs are associated with the triple negative subtype, whereas *BRCA2* GPVs are likely to result in higher grade, estrogen-receptor positive BCs. The contribution of other genes of high and moderate risk for BC has not been well defined and risk estimates to specific BC subtypes is lacking, especially for an admixed population like Brazilian.

**Objective:**

The aim of this study is to evaluate the value of a multigene panel in detecting germline mutations in cancer-predisposing genes for Brazilian BC patients and its relation with molecular subtypes and the predominant molecular ancestry.

**Patients and methods:**

A total of 321 unrelated BC patients who fulfilled NCCN criteria for *BRCA1/2* testing between 2016-2018 were investigated with a 94-genes panel. Molecular subtypes were retrieved from medical records and ancestry-specific variants were obtained from off-target reads obtained from the sequencing data.

**Results:**

We detected 83 GPVs in 81 patients (positivity rate of 25.2%). Among GPVs, 47% (39/83) were identified in high-risk BC genes (*BRCA1/2, PALB2* and *TP53*) and 18% (15/83) in moderate-penetrance genes (*ATM, CHEK2* and *RAD51C*). The remainder of the GPVs (35% - 29/83), were identified in lower-risk genes. As for the molecular subtypes, triple negative BC had a mutation frequency of 31.6% (25/79), with predominance in *BRCA1* (12.6%; 10/79). Among the luminal subtypes, except Luminal B HER2-positive, 18.7% (29/155) had GPV with *BRCA1/2* genes contributing 7.1% (11/155) and non-*BRCA1/2* genes, 12.9% (20/155). For Luminal B HER2-positive subtype, 40% (16/40) had GPVs, with a predominance of *ATM* gene (15% - 6/40) and *BRCA2* with only 2.5% (1/40). Finally, HER2-enriched subtype presented a mutation rate of 30.8% (4/13) with contribution of *BRCA2* of 7.5% (1/13) and non-*BRCA1/2* of 23% (3/13). Variants of uncertain significance (VUS) were identified in 77.6% (249/321) of the patients and the number of VUS was increased in patients with Asian and Native American ancestry.

**Conclusion:**

The multigene panel contributed to identify GPVs in genes other than *BRCA1*/*2*, increasing the positivity of the genetic test from 9.6% (*BRCA1/2*) to 25.2% and, considering only the most clinically relevant BC predisposing genes, to 16.2%. These results indicate that women with clinical criteria for hereditary BC may benefit from a multigene panel testing, as it allows identifying GPVs in genes that directly impact the clinical management of these patients and family members.

## Introduction

Breast cancer is the most common non-cutaneous cancer, and according to the World Health Organization, it is the second leading cause of cancer death among women worldwide (1). Between 5-10% of BCs are attributed to inherited genetic variations mainly in two high-risk genes, *BRCA1* and *BRCA2*, associated to the hereditary breast and ovarian cancer syndrome (HBOC), which confer a high risk of breast, ovarian, pancreatic and prostate cancer (2–4). However, a significant proportion of the suspected genetic risk patients remains unexplained when only the two genes, *BRCA1/2*, are investigated. Apart from *BRCA1/2*, GPVs located in seven other genes - *ATM, BARD1, CHEK2, PALB2, RAD51C, RAD51D* and *TP53 –* have shown to be clinically relevant, increasing the risk to develop BC (5–9). For *ATM, CHEK2* and *PALB*2 appropriate evidences have been gathered in the clinical setting to warrant the screening for GPVs in these genes, even in the absence of familial BC history (10–12). Additionally, the role of widespread clinical testing for GPVs in other BC-risk genes, such as *CDH1, STK11* and *PTEN* that increase the risk for BC in the context of Hereditary Diffuse Gastric Cancer, Peutz-Jeghers and Cowden’s syndrome, respectively, continues to be debated (9).

The implementation of genetic testing for multiple genes for hereditary cancer syndromes offers many benefits, including lower cost and time per gene when compared to single-gene testing (13–16). Currently available commercial multigene panels range widely from phenotype specific, for familial cancer such as BC, to panels covering multiple phenotypes. These panels may include high-risk genes, with established clinical utility, as well as moderate and low-risk genes, with limited data about clinical significance and cancer risk and even genes with no management guidelines (16, 17).

Important issues have been widely discussed about the clinical indications, benefits and genetic counseling impact of multigene panels (18). In general, these panels are indicated when more than one gene may be associated to the phenotype, due to its increased efficacy and reasonable cost as compared to single genes (16). Indications can also be considered for patients with a negative test for particular syndromes, whose personal and familial history may suggest hereditary cancer (13, 19). Nevertheless, nowadays, National Comprehensive Cancer Network (NCCN) guidelines recommend high-penetrance susceptibility gene analysis, beyond *BRCA1/2*, for BC patients with testing criteria (20).

The use of multigene panels in the clinical practice still faces some challenges. These include the proper interpretation of the sometimes complex results, such as the identification of variants of uncertain significance (VUS), specially concerning in populations that have been less characterized by genomic studies, as well as the find of unexpected results like variants without genotype-phenotype correlation and of potentially GPVs in moderate and low-risk genes, which makes genetic counseling and clinical management more challenging (11, 16, 17). As an example, after the implementation of multigene panels in suspected BC risk patients, some studies reported that the identification of at least one VUS in different cohorts varied from 20% to 40% (13, 19). Still, studies with multigene panels have shown that patients with suspected hereditary breast and ovarian cancer and negative for *BRCA1/2*, presented a prevalence of mutations in other genes ranging from 4% to 16%, substantially increasing the ability to discovery the genetic cause for the increased cancer risk in these patients (18, 19).

The aim of the study presented here is to evaluate the impact of the use of a multigene panel in clinical practice of patients suspected of BC risk in both, overall and subtype-specific BC scenarios, and to evaluate the VUS repertoire in groups distinct predominant ancestries. To this end, we used a 94-genes panel in a series of BC patients and compared the identified clinically relevant variants to clinical, pathological and ancestry data. Our results contribute to the understanding of the genetic architecture of BC risk in a very admixed and scarcely genetic characterized population.

## Methods

### Patients selection

We selected a total of 321 unrelated patients diagnosed with BC, all under investigation at the Department of Oncogenetics, A.C.Camargo Cancer Center (ACCCC), between September 2016 and May 2018. Inclusion criteria: patients with a current or previous BC diagnosed at any age, of any histological type (including bilateral BC), who fulfilled NCCN criteria (2016 to 2018) of HBOC syndrome and performed genetic test at the Genomic Diagnostic Laboratory/Pathological Anatomy of the ACCCC. All patients received pre- and post-testing genetic counseling.

All patients have signed a written informed consent after genetic counseling. This study was approved by the local Ethics Committee of the ACCCC (protocol number 2483/18).

### Genetic testing

Genomic DNA from peripheral blood sample or saliva was extracted and was used to perform capture by hybridization of the exons and exon-intron boundaries of the 94 genes using the commercial kits TruSight Cancer Sequencing Panel and TruSight Rapid Capture (Illumina). Next generation sequencing (NGS) was performed on the NextSeq 500 System (Illumina) platform. Sequences corresponding to the requested genes of each patient were compared with the respective reference sequences for calling variants using bioinformatics tools (Isaac Enrichment v3.0 and Illumina Variant Studio 2.2).

All identified variants were imported into the VarSeq software (Golden Helix) for function, classification and frequency annotations in public databases. Variants were filtered according to the criteria: quality >30; variant base present in at least 25% of the reads; absent in population databases (gnomAD, dbSNP, 1000genomes and Abraom - database of variants of exomes of the Brazilian population: http://abraom.ib.usp.br/) or, when present, presenting minor allele frequencies (MAF) ≤ 0.01.

Multiplex Ligation-dependent Probe Amplification (MLPA – P087, MRC-Holland, Amsterdam, NL) was used for *BRCA1* and *BRCA2* copy number variation analysis, according to the manufacturer’s recommendations. Coffalyzer software (MRC-Holland, Amsterdam, NL) was used at default settings for data analyses.

### Variant classification and analysis

The variants were noted for their increased changes of impacting protein function: loss of function (LoF) changes, indels and mutations at canonical splice sites; indels disrupting reading frames; amino-acid substitution variants (missenses) and synonymous alterations, and evaluated in the ClinVar database. For the classification and final interpretation of the identified variants we have followed the recommendations of the American College of Medical Genetics and Genomics (ACMG) (21).

### Ancestry analysis

Analysis of African, European, Native/Latin American, and South/East Asian ancestries were performed using sequencing data obtained from the TruSight Cancer, containing data from the 94 cancer predisposing genes as well as all off-target reads. The data was processed using the software PLINK and a set of quality control criteria was applied (22). First, SNPs with call rates across samples < 95% and minor allele frequency (MAF) < 1% were filtered out. Then, SNPs were excluded for Hardy-Weinberg Equilibrium test < 0.000001 and pruned for linkage disequilibrium for window size = 50, step size = 5 and r2threshold = 0.2. For quality control, the set of selected SNPs was applied to the reference dataset and the unsupervised mode of ADMIXTURE was used. The values obtained were then compared with the ancestry previously calculated. This comparison was made using the graphical method Bland-Altman (23). As a reference, populations from 1000 Genomes Project (1000G) and Human Genome Diversity Project (HGDP) were extracted (24–26). Altogether eight populations were selected from 1000G and these were grouped into four superpopulations: European, African, Native American and Asian. Only the most homogenous populations were selected. This estimate was obtained using the unsupervised mode of ADMIXTURE with K=4 (27). The chosen populations were: YRI (Yoruba in Ibadan, Nigeria), LWK (Luhya in Webuye, Kenya), GBR (British in England and Scotland), TSI (Toscani in Italia), JPT (Japanese from Tokyo, Japan), CHB (Han Chinese from Beijing, China), ITU (Indian Telugu from UK) and STU (Sri Lankan Tamil from the UK). In HGDP, five populations were selected, and classified as Native Americans. From all our subjects, a total of 534,734 SNPs were found.

The classification in categorical variables was performed similarly to two previous studies (28, 29). Individuals that had the secondary ancestry with less than 20% were noted as the first ancestry (e.g., an individual with 78% European ancestry, 15% African and 7% native American was annotated as predominantly EUR). Individuals that had greater than 20% of the secondary ancestry were classified as admixed samples, and both the primary and secondary most relevant ancestries were noted (e.g., an individual with 65% European ancestry and 35% African ancestry was annotated as EUR_admixAFR). Individuals without any ancestry above 50% were noted as highly admixed.

### Statistical analysis

Clinical, anatomopathological and familial characteristics were described with descriptive statistics, including medians, means and standard deviations for continuous data. For categorical data, proportions with a 95% confidence interval (CI) were calculated using the Clopper-Pearson method. The demographic, clinical and pathological data were compared by the T test (continuous variables) and the T test/analysis of variance for continuous variables. Statistical significance was set at a *p* ≤ 0.05.

Breast cancer (BC) molecular subtypes were classified according to Immunohistochemistry (IHC) status of Progesterone/Estrogen receptor and HER2 protein overexpression by IHC/amplified by FISH. Ki67 status was not available in our records. Thus we classified BC in three molecular subtypes: Triple-negative breast cancer (TNBC), Luminal (HR-positive/HER2-negative), Luminal B HER2 (HR-positive/HER2-positive) and HER2-enriched (HR-negative/HER2-positive).

To investigate the predictors of number of VUS, we used a multivariate poison regression. First set number of VUS as response variable and used all predictors (“European”, “African”, “Asian”, “America”, “Ashkenazi Jewish ethnicity”, “Age at diagnosis years”, “ Family history of breast & ovarian cancer”, “ Family history of non-breast & ovarian cancer”) in building a model.

In order to be able to select the features, which could well serve as predictors, we used backward stepwise approach where the least significant variable is gradually eliminated until we get a final model with relatively lower AIC values. To take a more robust approach, we bootstrapped the backward stepwise elimination approach running it for (n) number of times each time with subset of (n) samples from original dataset with replacement. We repeated the process with a) all patients are selected, b) selected patients with VUS in genes that form top 10% in the cohort, c) patients with VUS of 10 breast and ovarian cancer genes previously reported (30). For GPVs, we used a multivariate binomial logistic regression model of all predictors followed by bootstrap backward stepwise feature selection process. The response variable in this exercise was the presence or absence of GPVs.

## Results

### Germline characterization in breast cancer patients with overall or specific subtype

Among 321 women included, the mean age at diagnosis was 45.2 years (ranging from 26 to 85 years), and the median was 44 years, just over half of them developed BC at age 45 years or younger (57%) and 29 (9%) had bilateral disease. Only 1.2% of the studied population declared to have Ashkenazi Jewish ancestry. The predominant histological type was ductal carcinoma, found in 82.6% of the cases. Triple-negative breast cancer (TNBC) was found in 24.6% of the patients, Luminal subtype in 48.3%, Luminal B HER2 in 12.5%, HER2-enriched in 4.0%. Tumor subtype data was not available for 10.6% (**Table 1**). We observed that 12.1% of women had diagnosis of another cancer, and of these, 2.8% had a diagnosis of ovarian cancer (5 of them had diagnosis of ovarian after BC, one was synchronized and two had ovary before BC). Most women (65.1%) had a first, second or third degree relative with breast or ovary cancer. Clinical and pathological findings for all patients are given in **Table 1**.

**Table 1:** Clinical and anatomopathological characteristics found in the 321 studied individuals.

Study Characteristic (N = 321)	No.	%
**Age at diagnosis, years**		
Mean ± SD	45,21 ± 11,22	
Median	44	
Range	26-85	
≤45	183	57.0
46-60	106	33.0
>60	32	10.0
**Ashkenazi Jewish ethnicity**		
Yes	4	1.2
No	317	98.8
**Breast cancer subtypes, receptor status**		
TNBC	79	24.6
HR positive/HER2 negative (Luminal)	155	48.3
HR negative/HER2 positive (HER2- enriched)	13	4.0
HR positive/HER2 positive (Luminal B HER2)	40	12.5
HR positive/HER2 not available	22	6.8
Unknown	12	3.7
**Histology**		
Ductal	265	82.6
Lobular	27	8.4
Ductal and lobular	2	0.6
Other	20	6.2
Unknown	7	2.2
**Bilateral disease**		
Yes	29	9.0
No	292	91.0
**Patient history of second breast cancer**		
Yes	38	11.2
No	283	88.2
**Patient history of prior cancer (excluded breast cancer)**		
Yes	39	12.1
No	282	87.9
*Ovarian cancer*	9	2.8
**First-/second-/third- degree relative with breast or ovarian cancer**		
Yes	209	65.1
No	106	33.0
Unknown	6	1.9
**First-/second-/third- degree relative with cancer (excluded breast and ovarian)**		
Yes	244	76.0
No	71	22.1
Unknown	6	1.9

SD, standard deviation; TNBC, triple-negative breast cancer; HR, hormone receptor; HER2, human epidermal growth factor receptor 2.

Using a 94-genes panel a total of 83 GPVs in 24 genes were found in 81 women (25.2%). Most GPVs were LoF (78.3%): 32 frameshift variants (38.6%), 18 nonsense variants (21.7%) and 10 splice site variants (12%), and 21.7% were missense variants. The frequency of GPVs among 321 women was 25.2% (81/321) (**Table 2**). Two patients had more than one GPV and were diagnosed with the Multilocus Inherited Neoplasia Alleles Syndrome (MINAS), involving a combination of mutations in *BRCA2*/*ATM* and *BRCA2*/*CHEK2* genes.

**Table 2:** Frequency of germline pathogenic variants found in the study population.

Gene	No. of Patients	%	95% CI
**Negative for GPV**	240	74.8	
**Positive for GPV**	81	25.2	
**Total of GPV**	83		
**High risk breast cancer genes**	39	47.0	
*BRCA1*	17	20.5	12.4 – 30.8
*BRCA2*	14	16.9	9.5 – 26.7
*TP53*	4	4.8	1.3 – 11.9
*PALB2*	4	4.8	1.3 – 11.9
**Moderate risk breast cancer genes**	15	18.0	
*ATM*	8	9.6	4.3 – 18.1
*CHEK2*	6	7.2	2.7 – 15.1
*RAD51C*	1	1.2	0.0 – 6.5
**Low risk breast cancer genes**	29	35.0	
*MUTYH* (monoallelic)	7	8.4	3.5 – 16.6
*SBDS*	3	3.6	0.8 – 10.2
*FANCI*	2	2.4	0.3 – 8.4
*HNF1A*	2	2.4	0.3 – 8.4
*PFR1*	2	2.4	0.3 – 8.4
*RECQL4*	2	2.4	0.3 – 8.4
*BLM*	1	1.2	0.0 – 6.5
*BRIP1*	1	1.2	0.0 – 6.5
*FANCA*	1	1.2	0.0 – 6.5
*FANCD2*	1	1.2	0.0 – 6.5
*FANCE*	1	1.2	0.0 – 6.5
*FANCL*	1	1.2	0.0 – 6.5
*FANCM*	1	1.2	0.0 – 6.5
*FH*	1	1.2	0.0 – 6.5
*PHOX2B*	1	1.2	0.0 – 6.5
*PMS2*	1	1.2	0.0 – 6.5
*SLX4*	1	1.2	0.0 – 6.5

GPV, germline pathogenic variants.

Genes included in the multigene panel: AIP, ALK, APC, ATM, BAP1, BLM, BMPR1A, BRCA1, BRCA2, BRIP1, BUB1B, CDC73, CDH1, CDK4, CDKN1C, CDKN2A, CEBPA, CEP57, CHEK2, CYLD, DDB2, DICER1, DIS3L2, EGFR, EPCAM, ERCC2, ERCC3, ERCC4, ERCC5, EXT1, EXT2, EZH2, FANCA, FANCB, FANCC, FANCD2, FANCE, FANCF, FANCG, FANCI, FANCL, FANCM, FH, FLCN, GATA2, GPC3, HNF1A, HRAS, KIT, MAX, MEN1, MET, MLH1,, MSH2, MSH6, MUTYH, NBN, NF1, NF2, NSD1, PALB2, PHOX2B, PMS1, PMS2, PRF1, PRKAR1A, PTCH1, PTEN, RAD51C, RAD51D, RB1, RECQL4, RET, RHBDF2, RUNX1, SBDS, SDHAF2, SDHB, SDHC, SDHD, SLX4, SMAD4, SMARCB1, STK11, SUFU, TMEM127, TP53, TSC1, TSC2, VHL, WRN, WT1, XPA, XPC.

**Figure 1:**
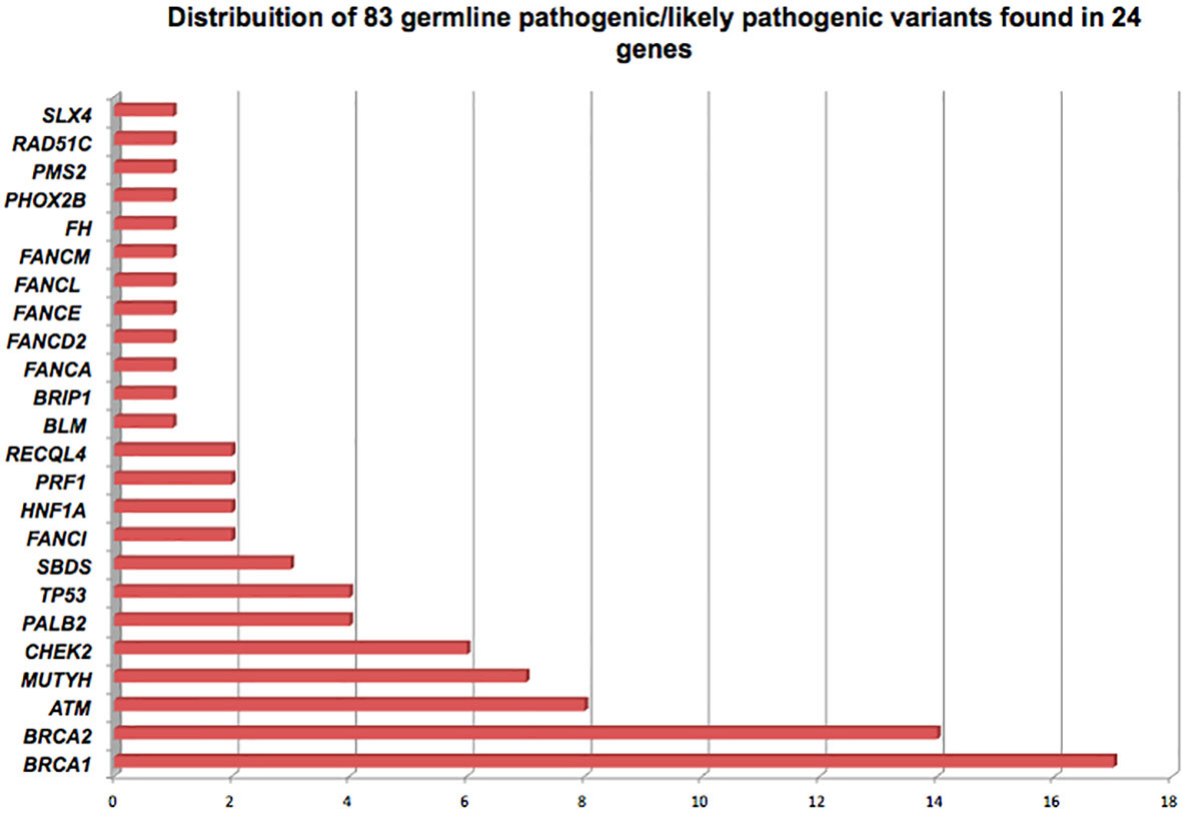
Distribution of 83 germline pathogenic/likely pathogenic variants of breast cancer-related genes detected in 81 Brazilian patients, found in 24 cancer susceptibility genes.

GPVs in genes of high-penetrance for BC were found in 39 patients (12.15% - 39/321) including: 17 (5.3%) in *BRCA1*; 14 in *BRCA2* (4.3%); 4 in *TP53* (1.2%) and 4 in *PALB2* (1.2%), corresponding to 47% (39/83) of the positive results. Mutations in genes of moderate penetrance for BC (*ATM, CHEK2, RAD51C*) were found in 4.6% (15/321) (**Figure 1**; **Table 2**).

*BRCA1/2* GPVs were found in 31 patients, corresponding to 9.6% (31/321) and to 37.3% (31/83) of the GPVs. In other 50 patients, corresponding to 15.6% (50/321), GPVs were detected in additional high, moderate and lower-risk BC genes: 23 GPVs (23/321 – 7.1%) were detected in high- and moderate-risk BC genes: 8 in *ATM* (9.6%), 6 with *CHEK2* (7.2%), 4 with *TP53* (4.8%), 4 with *PALB2* (4.8%), 1 with *RAD51C* (1.2%) and 29 GPVs (29/321 – 9%) in others of unknown clinical relevance for BC. (**Figure 1**; **Table 2**). No GPVs were identified in the other 70 genes. All GPVs detected are described in **Table 3**.

**Table 3:** Description of the identified 83 germline pathogenic variants.

ID	Gene	Chr:Pos	Type	HGVS Nomenclature	dbSNP	MAF (gnomAD)	Clinical Significance (Clinvar)	ACMGClassification
P017	*ATM*	11:108196153	Frameshift	c.6691_6692insCTTTT, (p.Leu2231SerfsTer6)	ND	ND	ND	Likely pathogenic
P047	*ATM*	11: 108196143	Missense	c.6679C>T, (p.Arg2227Cys)	rs564652222	ND	Pathogenic	
P062	*ATM*	11: 108138057	Nonsense	c.2626C>T, (p.Gln876Ter)	ND	ND	ND	Likely pathogenic
P087	*ATM*	11: 108155009	Frameshift	c.3802delG,(p.Val1268Terfs)	rs587779834	ND	Pathogenic	
P134	*ATM*	11: 108203613	Nonsense	c.7913G>A, (p.Trp2638Ter)	rs377349459	0.000017	Pathogenic	
P233	*ATM*	11: 108203613	Nonsense	c.7913G>A, (p.Trp2638Ter)	rs377349459	0.000017	Pathogenic	
P185	*ATM*	11: 108175549	Nonsense	c.5644C>T, (p.Arg1882Ter)	rs786204433	ND	Pathogenic	
P211	*ATM*	11:108192104	Nonsense	c.6529C>T, (p.Gln2177Ter)	rs766706861	0.0000039	ND	Likely pathogenic
P044	*BLM*	15: 91310153	Frameshift	c.2207_2212delinsTAGATTC, (p.Tyr736fs)	rs113993962	0.00017	Pathogenic	
P009	*BRCA1*	17: 41251790	Splice Donor	c.547+2T>A	rs80358047	ND	Pathogenic	
P012	*BRCA1*	17: 41244068 - 41244071	Frameshift	c.3477_3480delAAAG, (p.Ile1159Metfs)	rs80357781	ND	Pathogenic	
P018	*BRCA1*	17: 41256984	Intrônica	c.213-11T>G, IVS5-11T>G	rs80358061	0.000011	Pathogenic	
P065	*BRCA1*	17: 41219623	Splice Donor	c.5074+2T>C, IVS17+2T>C	rs80358089	ND	Pathogenic	
P082	*BRCA1*	17: 41246532	Frameshift	c.1016dupA (p.Val340GlyfsTer6)	rs80357569	ND	Pathogenic	
P123	*BRCA1*	17: 41199683	Nonsense	c.5444G>A, p.(Trp1815Ter)	rs80356962	ND	Pathogenic	
P131	*BRCA1*	17: 41219623	Splice Donor	c.5074+2T>C, IVS17+2T>C	rs80358089	ND	Pathogenic	
P132	*BRCA1*	17: 41209082	Frameshift	c.5266dupC, (p.Gln1756ProfsTer74)	rs80357906	ND	Pathogenic	
P145	*BRCA1*	17: 41215889	Splice Donor	c.5152+2T>C	rs886040914	ND	Pathogenic	
P154	*BRCA1*	17: 41243513	Frameshift	c.4035delA, (p.Glu1346LysfsTer20)	rs80357711	ND	Pathogenic	
P157	*BRCA1*	17: 41209082	Frameshift	c.5266dupC, (p.Gln1756ProfsTer74)	rs80357906	ND	Pathogenic	
P200	*BRCA1*	17: 41209082	Frameshift	c.5266dupC, (p.Gln1756ProfsTer74)	rs80357906	ND	Pathogenic	
P248	*BRCA1*	17: 41209082	Frameshift	c.5266dupC, (p.Gln1756ProfsTer74)	rs80357906	ND	Pathogenic	
P228	*BRCA1*	17: 41203112	Frameshift	c.5300delG, p.(Cys1767PhefsTer26)	ND	ND	ND	Likely pathogenic
P229	*BRCA1*	17: 41203112	Frameshift	c.5300delG, p.(Cys1767PhefsTer26)	ND	ND	ND	Likely pathogenic
P289	*BRCA1*	17: 41245861	Nonsense	c.1687C>T (p.Gln563Ter)	rs80356898	0.00004	Pathogenic	
P314	*BRCA1*	17: 41246251	Frameshift	c.1297delG, (p.Ala433Profs)	rs80357794	ND	Pathogenic	
P002	*BRCA2*	13: 32912236 - 32912239	Frameshift	c.3744_3747delTGAG, (p.Ser1248ArgfsTer10)	rs80359403	ND	Pathogenic	
P013	*BRCA2*	13:32914109 - 32914113	Frameshift	c.5617_5621delGTAAT, (p.Val1873Ter)	ND	ND	ND	Likely pathogenic
P052	*BRCA2*	13: 32914942	Frameshift	c.6450dupA, (p.Val2151SerfsTer25)	rs80359595	ND	Pathogenic	
P090	*BRCA2*	13: 32911300 - 32911303	Frameshift	c.2808_2811del, (p.Ala938ProfsTer21)	rs80359351	0.00002	Pathogenic	
P109	*BRCA2*	13:32953937	Missense	c.9004G>A, (p.Glu3002Lys)	rs80359152	ND	Pathogenic	
P130	*BRCA2*		Deletion	deletion éxons 1 e 2	ND	ND	ND	Likely pathogenic
P136	*BRCA2*	13: 32890599	Missense	c.2T>G, (p.Met1Arg)	rs80358547	0.00001	Pathogenic	
P211	*BRCA2*	13: 32893302 - 32893303	RNA splicing	c.156_157insAlu	ND	ND	Pathogenic	
P238	*BRCA2*	13: 32911300 - 32911303	Frameshift	c.2808_2811del, (p.Ala938Profs)	rs80359351	0.00002	Pathogenic	
P245	*BRCA2*	13: 32900635	Splice Acceptor	c.517-1G>A, IVS6-1G>A	rs81002849	ND	Pathogenic	
P250	*BRCA2*	13: 32929114	Nonsense	c.7124T>G, (p.Leu2375Ter)	rs886040687	ND	Pathogenic	
P275	*BRCA2*	13: 32915148	Nonsense	c.6656C>G, (p.Ser2219Ter)	rs80358893	ND	Pathogenic	
P309	*BRCA2*	13: 32972521	Frameshift	c.9871delT, (p.Ser3291Leufs)	rs886040854	ND	Pathogenic	
P319	*BRCA2*	13: 32914174	Nonsense	c.5682C>G, (p.Tyr1894Ter)	rs41293497	ND	Pathogenic	
P107	*BRIP1*	17: 59793412	Nonsense	c.2392C>T, (p.Arg798Ter)	rs137852986	0.00015	Conflicting	Pathogenic
P003	*CHEK2*	22: 29091857	Frameshift	c.1100delC, (p.Thr367Metfs)	rs555607708	0.00182	Conflicting	Pathogenic
P004	*CHEK2*	22: 29121326	Missense	c.349A>G, (p.Arg117Gly)	rs28909982	0.00011	Pathogenic	
P033	*CHEK2*	22: 29121326	Missense	c.349A>G, (p.Arg117Gly)	rs28909982	0.00011	Pathogenic	
P048	*CHEK2*	22: 29121058	Missense	c.499G>A, (p.Gly167Arg)	rs72552322	0.000023	Conflicting	Likely pathogenic
P105	*CHEK2*	22: 29091857	Frameshift	c.1100delC, (p.Thr367Metfs)	rs555607708	0.00182	Conflicting	Pathogenic
P013	*CHEK2*	22:29121087	Missense	c.470T>C, (p.Ile157Thr)	rs17879961	0.0049	Conflicting	Likely pathogenic
P261	*FANCA*	16:89862330	Frameshift	c.983_986TCAC, (p.His330AlafsTer4)	rs772359099	0.000042	Pathogenic	
P076	*FANCD2*	3:10133904	Nonsense	c.3817C>T, (p.Arg1273Ter)	rs745930696	0.000015	ND	Likely pathogenic
P208	*FANCE*	6:35423630	Nonsense	c.355C>T, (p.Gln119Ter)	rs121434505	0.000011	Pathogenic	
P096	*FANCI*	15: 89849381	Frameshift	c.3493delG, (p.Asp1165Thrfs)	rs1060501884	ND	Pathogenic	
P264	*FANCI*	15:89828432	Nonsense	c.1804C>T, (p.Arg602Ter)	rs1432325198	0.000010	ND	Likely pathogenic
P030	*FANCL*	2:58388668	Inframe	c.1007_1009delTAT, (p.Ile336_Cys337delinsSer)	rs747253294	ND	Conflicting	Likely pathogenic
P043	*FANCM*	14:45618145 - 45618161	Frameshift	c.865_881delCTTATTGTTCCGCTTGG, (p.Leu289Ter)	ND	ND	ND	Likely pathogenic
P042	*FH*	1:241661228	Inframe	c.1431_1433dupAAA, (p.Lys477dup)	rs367543046	0.0010	Conflicting	Likely pathogenic
P080	*HNF1A*	12:121432118	Frameshift	c.872dupC, (p.Gly292ArgfsTer25)	rs587776825	ND	Pathogenic	
P296	*HNF1A*	12:121432118	Frameshift	c.872dupC, (p.Gly292ArgfsTer25)	rs587776825	ND	Pathogenic	
P071	*MUTYH*	1: 45798475	Missense	c.536A>G, (p.Tyr179Cys)	rs34612342	0.0015	Pathogenic	
P192	*MUTYH*	1: 45798475	Missense	c.536A>G, (p.Tyr179Cys)	rs34612342	0.0015	Pathogenic	
P155	*MUTYH*	1: 45797228	Missense	c.1187G>A, (p.Gly396Asp)	rs36053993	0.003	Pathogenic	
P203	*MUTYH*	1: 45797228	Missense	c.1187G>A, (p.Gly396Asp)	rs36053993	0.003	Pathogenic	
P285	*MUTYH*	1: 45797228	Missense	c.1187G>A, (p.Gly396Asp)	rs36053993	0.003	Pathogenic	
P058	*MUTYH*	1:45797760	Splice Acceptor	c.934-2A>G	rs77542170	0.0011	Conflicting	Likely pathogenic
P147	*MUTYH*	1:45797760	Splice Acceptor	c.934-2A>G	rs77542170	0.0011	Conflicting	Likely pathogenic
P023	*PALB2*	16: 23641218	Nonsense	c.2257C>T, (p.Arg753Ter)	rs180177110	0.000023	Pathogenic	
P254	*PALB2*	16: 23649427	Frameshift	c.72delG, (p.Arg26Glyfs)	rs180177142	ND	Pathogenic	
P266	*PALB2*	16: 23637594	Nonsense	c.2711G>A, (p.Trp904Ter)	rs1060502726	ND	Pathogenic	
P304	*PALB2*	16: 23646627	Nonsense	c.1240C>T, (p.Arg414Ter)	rs180177100	0.0000079	Pathogenic	
P036	*PHOX2B*	4:41748030	Frameshift	c.739delG, (p.Ala247ProfsTer62)	ND	ND	ND	Likely pathogenic
P284	*PMS2*	7:6045549	Missense	c.137G>T, (p.Ser46Ile)	rs121434629	0.00017	Likely pathogenic	
P001	*PRF1*	10:72358804	Missense	c.673C>T; (p.Arg225Trp)	rs28933973	0.000012	Pathogenic	
P166	*PRF1*	10:72358189	Frameshift	c.1288dupG, (p.Asp430GlyfsTer28)	rs1226526104	ND	ND	Likely pathogenic
P081	*RAD51C*	17:56798156	Frameshift	c.890_899del, (p.Leu297HisfsTer2)	rs1555602141	ND	Pathogenic	
P056	*RECQL4*	8:145741776	Nonsense	c.727C>T, (p.Gln243Ter)	rs1345625725	0.000031	ND	Likely pathogenic
P313	*RECQL4*	8:145738437	Frameshift	c.2547_2548delGT, (p.Phe850ProfsTer33)	rs778141083	0.000010	Pathogenic	
P069	*SBDS*	7:66459197	Splice Donor	c.258+2T>C	rs113993993	0.0038	Pathogenic	
P112	*SBDS*	7:66459197	Splice Donor	c.258+2T>C	rs113993993	0.0038	Pathogenic	
P283	*SBDS*	7:66459197	Splice Donor	c.258+2T>C	rs113993993	0.0038	Pathogenic	
P230	*SLX4*	16:3633330	Frameshift	c.4921dupG, (p.Val1641GlyfsTer15)	rs770425994	0.000027	ND	Likely pathogenic
P097	*TP53*	17: 7574017	Missense	c.1010G>A, (p.Arg337His)	rs121912664	0.00001	Pathogenic	
P160	*TP53*	17: 7574017	Missense	c.1010G>A, (p.Arg337His)	rs121912664	0.00001	Pathogenic	
P277	*TP53*	17: 7574017	Missense	c.1010G>A, (p.Arg337His)	rs121912664	0.00001	Pathogenic	
P256	*TP53*	17:7577121	Missense	c.817C>T, (p.Arg273Cys)	rs121913343	0.00001	Pathogenic	

Chr:Pos, chromosome position; HGVS, Human Genome Variation Society; dbSNP, Single Nucleotide Polymorphism database; MAF, Minor allele frequency; gnomAD, genome aggregation database; ND, not described; ACMG, American College of Medical Genetics and Genomics.

**Figure 2:**
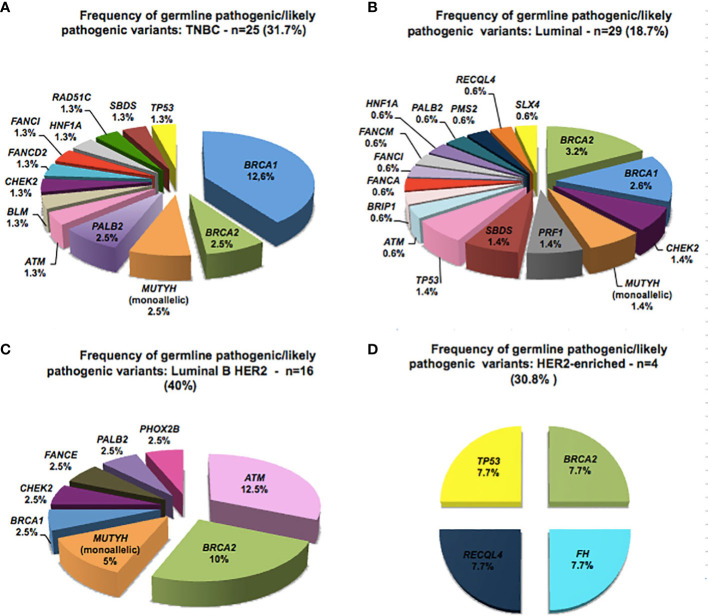
Spectrum of germline pathogenic variants detected according to the molecular subtype of breast cancer.

The presence of GPV was analyzed according to BC molecular subtypes (**Figure 2**). Among 79 TNBC patients, 31.6% (26/79) had a GPV and the gene with the highest number of GPVs was *BRCA1* (12.6%) followed by *BRCA2*, *MUTYH* and *PALB2* (2.5% each), and *TP53* and *RAD51C* had 1.3% each. Luminal BC was diagnosed in 155 patients, and of these, 18.7% (29/155) had a P/LP variant, with *BRCA2* and *BRCA1* showing 5.8% of mutations (3.2% and 2.6%, respectively). The subtype Luminal B HER2 was found in 40 patients, and of these, 40% (16/40) had a P/LP variant, with *BRCA2* mutation being found in 10% (4/40) and *ATM* in 12.5% (5/40), the most frequently mutated gene in this subgroup. Finally, for the 13 patients diagnosed with HER2-enriched BC, 30.8% (4/13) had a P/LP variant in *BRCA2, TP53, FH* and *RECQL4* genes (7.7% each), these latter two genes with an still to be determined relevance in BC (**Figure 2**).

**Figure 3:**
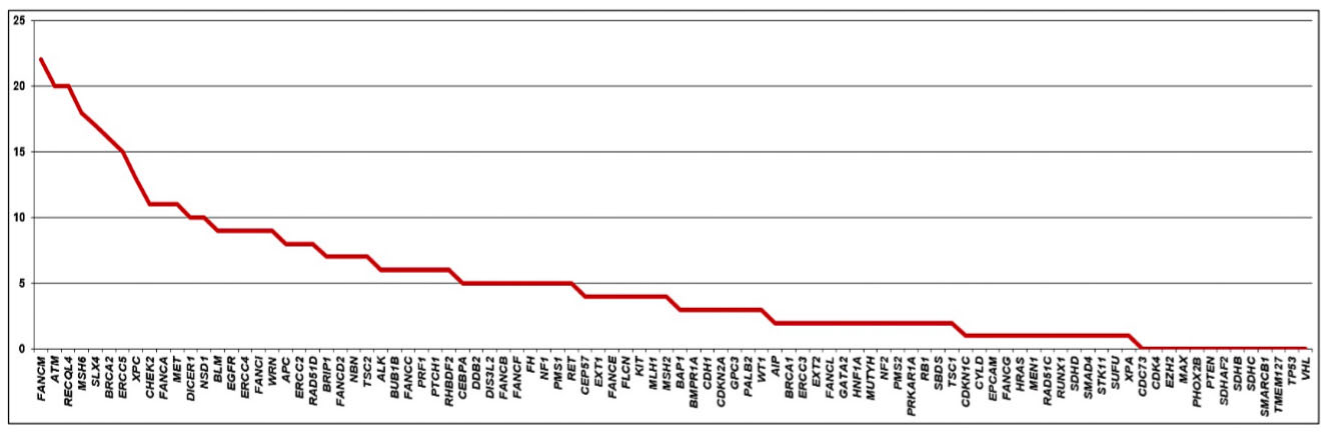
Distribution of variants of uncertain significance, according to the identified gene.

At least one VUS was identified in 249 patients (249/321 - 77.5%). A total of 470 variants were found in 81 of the 94 genes of the panel. *FANCM* gene harbored the larger number of VUS, found in 22 patients (22/249 - 8.8%), followed by *ATM* and *RECQL4*, with VUS identified in 20 patients each (20/249 - 8%), *MSH6* with VUS identified in 18 patients (18/249 - 7.2%), *SLX4* in 17 patients (17/249 - 6.8%) and *BRCA2* in 16 (16/249 – 6.4%). Some patients had more than one VUS in the same gene (**Figure 3**). Most VUS were identified in genes without strong evidence of association with breast cancer.

Two patients were diagnosed with MINAS, the first (P013) was diagnosed with a ductal carcinoma at the age of 49 years, molecular subtype luminal B HER2 with family history of breast, prostate, colorectal and gastric cancer. For this subject we detected the GPV c.5617_5621delGTAAT; p.(Val1873*) in *BRCA2* gene and the variant c.470T>C; p.(Ile157Thr) in *CHEK2* gene. The variant c.470T>C is a founder variant with low penetrance in Finnish and Polish individuals (31, 32). Mutations in these two genes increase the risk for BC and *BRCA2* is also related to increase risk for prostate cancer even as *CHEK2* increases risk for colorectal cancer, probably explaining the family history of cancer at multiple distinct sites.

The second patient (P211) was diagnosed with a ductal carcinoma at 31 years old, molecular subtype luminal B HER2 with a family history of BC in a second-degree relative. The GPV c.156_157insAlu was detected in *BRCA2* gene and the variant c.6529C>T; p.(Gln2177Ter) in *ATM* gene, both genes related to BC.

We evaluated potential associations among clinical variants and the BC carriers with GPVs in both *BRCA1* and *BRCA2* genes, and 22 additional genes (*ATM, BLM, BRIP1, CHEK2, FANCA, FANCD2, FANCE, FANCI, FANCL, FANCM, FH, HNF1A, MUTYH, PALB2, PHOX2B, PMS2, PRF1, RAD51C, RECQL4, SBDS, SLX4* and *TP53*). Significant associations were found only with *BRCA1/2* GPVs and self-reported Ashkenazi Jewish ethnicity. No other significant associations were found with the other clinical variables evaluated (age of onset, BC histology, hormone receptor status, bilateral BC, personal history of other malignant neoplasms and familial history of cancer) (**Supplementary Table 1**) neither in the additional 22 genes with GPVs (**Supplementary Table 2**).

Among patients without a GPV, the mean age at diagnosis of BC was 45.5 years. For patients with GPVs, the mean age at diagnosis was 44.2 years for *BRCA1*, 42.8 for *BRCA2*, 46.7 for *TP53*, 38.1 for *ATM* and 40.2 for *PALB2* carriers. Mean age in patients with GPVs located in low risk genes for BC was 46.3 years. Among patients diagnosed with BC before 45 years, 25.6% (47/183) had a GPV; between ages 46 and 60 years, 29.2% (31/106) and after 60 years of age, only 9.3% (3/32).

Regarding the diagnosis of other malignancies, excluding BC, 12.1% (39/321) of the women included had a diagnosis of another primary cancer and the most frequent was ovarian cancer (9/39 - 23%), followed by thyroid (7/39 - 18%) and colorectal cancer (7/39 - 18%).

### Ancestry analysis

Genetic variants in target and off-target regions captured by the multigene panel were used to access the genetic ancestry of our cohort of 321 non-related women with BC (**Supplementary Figure 1**). According to the proportion of first and second most common ancestries, patients were divided into eleven categories. Most patients were classified as having predominant European ancestry (183/321 – 57.0%), or European admixed with a second ancestry (51/321 – 15.9%). African ancestry was observed as the major ancestry in only 12 patients (3.7%) and as the second ancestry in 36 patients (11.0%); and finally Asian ancestry was observed as major ancestry in 16 patients (5.0%) and Asian/Native American as second ancestries in 0.9% (3/321) (**Supplementary Table 3**).

As molecular and cancer genomics studies are sparse for populations, such as those with African, as well as Asian/Native American ancestries we hypothesized that more VUS would be found in among non-Europeans. Also, if these indeed represent real cancer risk, some variations could have stronger associations with familial cancer history or early age at diagnosis. We therefore investigated whether family history of cancer, age at diagnosis, ancestry and ethnicity may be associated with number of VUS per patient. Using multivariate Poisson regression with feature selection process (see methods) we found evidence for Asian ancestry and family history (1^st^, 2^nd^ and 3^rd^ degree relatives) with any cancer other than breast and ovarian cancer as positive predictors of the number of VUS. For an individual with family history of cancer other than breast and ovarian cancer, their incidence rate ratios of having VUS is 1.35 [95% CI 1.06 – 1.75, p = 0.019] when all other variables are held constant. With a percentage increase in Asian and Native American ancestry we would expect number of VUS to increase by a factor of 1.61 [95% CI 1.09 – 2.32, p = 0.013] and 4.58 [95% CI 1.30 – 13.76, p = 0.011] respectively per increase in percent (**Supplementary Table 4**). However, incidence ratios were higher when narrowed down to patients, who had VUS in any of the eight frequently mutated genes (top 10% VUS) in the series thus *FANCM, ATM, MSH6, RECQL4, BRCA2, ERCC5, SLX4* and *XPC*.

Regarding GPVs, using a multivariate binomial logistic regression model of all predictors followed by bootstrap backward stepwise feature selection process, we found that only the Ashkenazi Jewish ethnicity had positive association with presence or absence of GPVs. The odds ratio of having GPVs was 9.19 [95% 1.16 – 187.30] but reached no statistical significance (p = 0.056) (**Supplementary Table 5**). There was no difference between the ancestries groups and the frequency of GPV in the most frequent mutated genes (*BRCA1, BRCA2, TP53, PALB2, ATM, CHEK2* and *MUTYH*). Breast cancer molecular subtypes (TBNC and non-TNBC) and age of tumor onset were also similar among different ancestry categories (data not shown).

## Discussion

The use of multigene panels for genetic counselling in hereditary BC is growing more and more. However, although the use of these panels can help in the diagnosis cancer predisposition syndromes for some families, the challenges of interpreting the results for meaningful genetic counseling still lingers, as cancer risk estimates and management strategies still have to be established for many genes.

In the present study, among 321 unrelated women with BC, the frequency of GPVs in *BRCA1/2* genes was 9.6% and in non-*BRCA1/2* cancer predisposition genes was 15.6%. Overall the analysis of the 94-genes panel contributed to identify GPVs in non-*BRCA1/2* in 50 patients, increasing the frequency of variants identification by almost 16%, similar to some previous studies in BC (33–36). Considering the 9 BC-genes, which were recently described as the most relevant BC predisposing genes (*BRCA1, BRCA2*, *ATM, BARD1, CHEK2, PALB2, RAD51C, RAD51D* and *TP53*) (9), 16.8% patients were detected as carriers GPV in at least one of these genes. The seven non-*BRCA1/2* genes contributed with 7.2% in the ability to detect a GPV as a genetic determinant of BC in these women, showing a main gain in terms of clinical value in analyzing of these 9 BC-genes instead of only *BRCA1/2*.

Our findings demonstrated a higher prevalence of GPVs in high-risk BC genes, such as *BRCA1*, *BRCA2*, *PALB2* and *TP53* as expected. Pathogenic variants in *TP53* were identified in four patients and three of them harbored the same variant - c.1010G>A (p.Arg337His/R337H), that was introduced in Brazil possibly a founder effect, and is now found in relatively high frequencies in the southeast and southern regions of the country (37). A recent study identified a variant in the tumor suppressor *XAF1* (E134*) in a subset of R337H carriers and proposes that the co-segregation of *XAF1*-E134* and *TP53*-R337H mutations leads to a more aggressive cancer phenotype than R337H alone (38). The analysis of variant E134* was positive in 2 patients (P097 and P277) and negative in patient P160. No GPV were identified in the other high-risk BC genes such as *PTEN*, *CDH1* and *STK11* and they are very rare, as demonstrated in a recent study (9).

As for variants of moderate risk for BC, GPVs in *ATM*, *CHEK2* and *RAD51C* were found in 4.6% of our series and corresponding to 18% of GPVs found, a finding consistent with other recent studies (33–36, 39). According to Tung et al. (2016), germline mutations in moderate risk BC susceptibility genes are identified in approximately 2% to 5% of individuals performing multigene panel (36). It is important to note that, after *BRCA1/2, ATM* was the most prevalent mutated gene among patients in our study. De Souza Timoteo et al. (2018) reported that germline mutations in moderate- and low-risk BC genes were detected in 3.8% of individuals, including *ATM, ATR, CDH1, MLH1* and *MSH6* (40).

Our results are similar to other Brazilian studies. De Souza Timoteo et al. (2018) evaluated 157 individuals (132 with breast and 25 cancer-unaffected) using three different types of multigene panel (40). Germline pathogenic variants were identified in twenty-seven individuals (17.2%), 24 with BC and three asymptomatic, and most of them in *BRCA1*/*2* genes (75%) (38). A recent study evaluated germline molecular data in hereditary BC in 224 patients and GPVs were detected in 20.5% (41). The frequency of GPV in a high-penetrance BC gene was 61% and frequency of moderate penetrance genes represented 15.2% of the positive results (41).

According to the guidelines of the NCCN, screening recommended for patients with moderate-risk BC GPVs such as *ATM* and *CHEK2* is annual mammogram and consider breast MRI with contrast due to increased risk of BC (20). There is insufficient evidence for risk-reducing mastectomy and should be based on family history. In our series, just one of the patients with a GPV in *CHEK2* had a contralateral BC and none of the patients with a GPV in the moderate-risk BC genes had other primary cancers (excluding BC) (20). Bilateral BC was not significantly associated with GPVs in our cohort.

Regarding the molecular subtype of BC, we observed the predominance of GPVs in *BRCA1* genes in TNBC tumors, as reported by others, including our own previous study with 131 Brazilian women with TNBC (42–45). For the luminal subtype, GPVs in *BRCA1* and *BRCA2* genes were found in only 5.8%, while GPVs in other genes correspond to 12.9%, highlighting the contribution of the multigene panel in luminal tumors. Moreover, we found that the proportion of *ATM* GPVs is significantly higher in Luminal B HER2 tumors, as previously reported in the literature (46).

Seven carriers with GPV in low-risk gene *MUTYH* (monoallelic), were found here, whose association with BC risk is still controversial. Some studies reported an increased risk of BC for monoallelic *MUTYH* mutation carriers (47, 48). However, other studies did not find statistical evidence for an increased risk of BC (49, 50).

Recently, it has been described the MINAS condition, which is characterized by the presence of two or more GPVs in genes related to cancer predisposition in the same individual (51). We found a frequency of 2.4% among patients who are carriers of two GPVs, with the following combinations *BRCA2*/*ATM* and *BRCA2*/*CHEK2*. An overlap of phenotypes associated with both genes was observed in theses cases.

According to previous studies, about a third of multigene panels identify at least one VUS in one or more genes (18, 19). A study of 2,158 women with BC referred to genetic testing using a 25-multigene panel, showed that VUS were found in 40% of individuals (19). Another similar study with 198 women who underwent 42 multigene panel showed that VUS was identified in 88% of them (18). In our series using a 94-genes panel, we found 77.5% of patients with VUS and showed that patients with Asian and Native American ancestry were associated to a higher number of VUS. It is expected that the panels containing a larger number of genes will result in a higher rate of patients presenting VUS. Also, as most VUS represent rare missense variants with low minor allele frequencies or not described in populational databases, it is anticipated that genetically less characterized populations, such as the Brazilian, will have more VUS. In this sense, a recent study evaluating more than a 100,000 multigene hereditary cancer genetic tests revealed that, compared to Europeans, Asian and Middle Eastern individuals were most likely to be identified with VUS (52).

It should be noted that there were limitations associated with our study. Copy number variation analysis with MLPA was only performed for *BRCA1/2*. The panel used is not validated for large deletion/duplication analysis. Patients selected to perform the multigene panel had criteria for HBOC, with personal or family history that suggested higher inherited cancer risk. At the same time, it is possible that we did not include BC patients who did not meet criteria for HBOC but could have GPVs in the other genes included in the panel. Another limitation of the present study was that we could not establish a genotype-phenotype correlation for moderate and low-risk BC genes, due to the small number of patients with GPVs in these genes.

In conclusion, our results indicate that although most of the GPV found in this study were in the *BRCA1/2* genes (9.6%), women who fulfill the clinical criteria for HBOC may benefit from multigene panel testing, because the panel allows to identify GPV in relevant BC predisposing genes (7.2%), including those who change the clinical management. This is the first study that analyzed multigene panel and its relation with molecular subtypes in Brazilian BC patients. Further studies are still needed to better comprehend the heritability of distinct subtypes of BC in Brazilian women, including those who do not fulfill clinical criteria for HBOC to correlate genotype-phenotype of moderate and low-risk BC genes.

## Data availability statement

The original contributions presented in the study are included in the article/**Supplementary Material**. Further inquiries can be directed to the corresponding author.

## Ethics statement

The studies involving human participants were reviewed and approved by Ethics Committee of the A. C. Camargo Cancer Center (protocol number 2483/18). The patients/participants provided their written informed consent to participate in this study.

## Author contributions

Conceptualization and design of the study: DP, MF, GT and DC; Data analysis: DP, GT, KS and DC; Carrying out of the Ancestry algorithm: EN and IS; Ancestry analysis: SA and PP; Writing and editing the original draft preparation: DP, GT, MF, PP, WF and DC; Funding acquisition, DC. All authors have read and agreed to the published version of the manuscript.

## Funding

This research was funded by Fundação de Amparo à Pesquisa do Estado de São Paulo, grant number 2014/50943-1, Conselho Nacional de Desenvolvimento Científico e Tecnológico, grant number 465682/2014-6 and Coordenação de Aperfeiçoamento de Pessoal de Nível Superior (CAPES) - 88887.136405/2017-00.

## Acknowledgments

We acknowledge the patients who participated in the study, and the A.C. Camargo Laboratory of Genomic Diagnostic.

## Conflict of interest

Author PP was employed by C2i Genomics.

The remaining authors declare that the research was conducted in the absence of any commercial or financial relationships that could be construed as a potential conflict of interest.

## Publisher’s note

All claims expressed in this article are solely those of the authors and do not necessarily represent those of their affiliated organizations, or those of the publisher, the editors and the reviewers. Any product that may be evaluated in this article, or claim that may be made by its manufacturer, is not guaranteed or endorsed by the publisher.
