# Supplementary material for: Characterization of genetic predisposition to molecular subtypes of breast cancer in Brazilian patients
Source: Front Oncol. 2022 Aug 31;12:976959. doi: 10.3389/fonc.2022.976959 (PMC9472814; doi:10.3389/fonc.2022.976959)
Supplement: Supplementary file 1 [file DataSheet_1.docx]

**Supplementary Material**

**Characterization of genetic predisposition to molecular subtypes of breast cancer in Brazilian patients**

Daniele Paixão, Giovana Tardin Torrezan, Karina Miranda Santiago, Maria Nirvana Formiga, Samuel Terkper Ahuno, Emmanuel Dias Neto, Israel Tojal da Silva, William D Foukes, Paz Polak, Dirce Maria Carraro.

Ancestry proportion

**Supplementary Figure 1:** Frequency of four major ancestry groups in 282 non-related Brazilian women with breast cancer. EUR: European; AFR: African; AMR: Native American; ASN: Asian.

**Supplementary Table 1: Clinical variables analyzed, looking for an association with germline pathogenic variants in the 24 genes of the panel. Only one GPV per patient was included.**

| Predictor | No GPV | | GPV | | *P* Value |
| --- | --- | --- | --- | --- | --- |
|  | **No.** | **%** | **No.** | **%** |  |
| Age at diagnosis (years) |  |  |  |  |  |
| ≤45 | 136 | 56.7% | 47 | 58.0 % | 0.07 |
| 46-60 | 75 | 31.3% | 31 | 38.3% |  |
| >60 | 29 | 12.1% | 3 | 3.7% |  |
| Ashkenazi Jewish ethnicity |  |  |  |  |  |
| Yes | 1 | 0.4% | 3 | 3.7% | 0.05 |
| No | 239 | 99.6% | 78 | 96.3% |  |
| Breast cancer subtypes, receptor status | |  |  |  |  |
| TNBC | 54 | 22.5% | 25 | 30.9% | 0.06 |
| HR positive/HER2 negative (Luminal) | 126 | 52.5% | 29 | 35.8% |  |
| HR negative/HER2 positive (HER2- enriched) | 9 | 3.8% | 4 | 4.9% |  |
| HR positive/HER2 positive  (Luminal B Her2) | 24 | 10.0% | 16 | 19.8% |  |
| HR positive/HER2 not available | 18 | 7.5% | 4 | 4.9% |  |
| Unknown | 9 | 3.8% | 3 | 3.7% |  |
| Histology |  |  |  |  |  |
| Ductal | 198 | 82.5% | 67 | 82.7% | 0.75 |
| Lobular | 19 | 7.9% | 8 | 9.9% |  |
| Ductal and lobular | 1 | 0.4% | 1 | 1.2% |  |
| Other | 16 | 6.7% | 4 | 4.9% |  |
| Unknown | 6 | 2.5% | 1 | 1.2% |  |
| Bilateral disease |  |  |  |  |  |
| Yes | 18 | 7.5% | 11 | 13.6% | 0.15 |
| No | 222 | 92.5% | 70 | 86.4% |  |
| Patient history of prior cancer (excluded breast cancer) | | | | | |
| Yes | 25 | 10.4% | 14 | 17.3% | 0.15 |
| No | 215 | 89.6% | 67 | 82.7% |  |
| First-/second-/third- degree relative with breast or ovarian cancer | | | | | |
| Yes | 155 | 64.6% | 54 | 66.7% | 0.5 |
| No | 79 | 32.9% | 27 | 33.3% |  |
| Unknown | 6 | 2.5% | 0 | 0% |  |
| First-/second-/third- degree relative with cancer (excluded breast and ovarian) | | | | | |
| Yes | 183 | 76.3% | 61 | 75.3% | 0.9 |
| No | 52 | 21.7% | 19 | 23.5% |  |
| Unknown | 5 | 2.1% | 1 | 1.20% |  |

Abbreviations: GPV: germline pathogenic variants; TNBC: triple-negative breast cancer; HR: hormone receptor; HER2: human epidermal growth factor receptor 2.

**Supplementary Table 2: Clinical variables analyzed, looking for an association with germline pathogenic variants in the 22 genes of the panel (excluding *BRCA1/2*). Only one GPV per patient was included.**

| Predictor | No GPV | | GPV | | *P* Value | |
| --- | --- | --- | --- | --- | --- | --- |
|  | **No.** | **%** | **No.** | **%** | |  |
| Age at diagnosis (years) |  |  |  |  | |  |
| ≤45 | 136 | 56.7% | 29 | 55.8% | | 0.32 |
| 46-60 | 75 | 31.3% | 20 | 38.5% | |  |
| >60 | 29 | 12.1% | 3 | 5.8% | |  |
| Ashkenazi Jewish ethnicity |  |  |  |  | |  |
| Yes | 1 | 0.4% | 2 | 3.8% | | 0.08 |
| No | 239 | 99.6% | 50 | 96.2% | |  |
| Breast cancer subtypes, receptor status | | | | | | |
| TNBC | 54 | 22.5% | 13 | 25.0% | | 0.1 |
| HR positive/HER2 negative (Luminal) | 126 | 52.5% | 20 | 38.5% | |  |
| HR negative/HER2 positive (HER2- enriched) | 9 | 3.8% | 3 | 5.8% | |  |
| HR positive/HER2 positive  (Luminal B Her2) | 24 | 10.0% | 13 | 25.0% | |  |
| HR positive/HER2 not available | 18 | 7.5% | 2 | 3.8% | |  |
| Unknown | 9 | 3.8% | 1 | 1.9% | |  |
| Histology |  |  |  |  | |  |
| Ductal | 198 | 82.5% | 43 | 82.7% | | 0.2 |
| Lobular | 19 | 7.9% | 7 | 13.5% | |  |
| Ductal and lobular | 1 | 0.4% | 1 | 1.9% | |  |
| Other | 16 | 6.7% | 1 | 1.9% | |  |
| Unknown | 6 | 2.5% | 0 | 0.0% | |  |
| Bilateral disease |  |  |  |  | |  |
| Yes | 18 | 7.5% | 4 | 7.7% | | 1,0 |
| No | 222 | 92.5% | 48 | 92.3% | |  |
| Patient history of prior cancer (excluded breast cancer) | | | | | | |
| Yes | 25 | 10.4% | 7 | 13.5% | | 0.69 |
| No | 215 | 89.6% | 45 | 86.5% | |  |
| First-/second-/third- degree relative with breast or ovarian cancer | | | | | | |
| Yes | 155 | 64.6% | 34 | 65.4% | | 0.76 |
| No | 79 | 32.9% | 18 | 34.6% | |  |
| Unknown | 6 | 2.5% | 0 | 0% | |  |
| First-/second-/third- degree relative with cancer (excluded breast and ovarian) | | | | | | |
| Yes | 183 | 76.3% | 42 | 80.8% | | 0.82 |
| No | 52 | 21.7% | 9 | 17.3% | |  |
| Unknown | 5 | 2.1% | 1 | 1.92% | |  |

Abbreviations: GPV: germline pathogenic variants; TNBC: triple-negative breast cancer; HR: hormone receptor; HER2: human epidermal growth factor receptor 2.

**Supplementary Table 3: Categorical classification of ancestry in 321 non-related Brazilian women with breast cancer**

| **Ancestry Classification** | **N** | **%** |
| --- | --- | --- |
| EUR | 183 | 57.0% |
| EUR_adAFR | 35 | 10.9% |
| EUR_adASN | 12 | 3.7% |
| EUR_adAMR | 4 | 1.2% |
| ASN | 16 | 5.0% |
| ASN_adEUR | 2 | 0.6% |
| AFR | 2 | 0.6% |
| AFR_adEUR | 9 | 2.8% |
| AFR_adAMR | 1 | 0.3% |
| AMR_adAFR | 1 | 0.3% |
| Admix | 17 | 5.3% |
| Not available | 39 | 12.1% |
| **Total** | **321** | **100.0%** |

EUR: European; AFR: African; AMR: Native American; ASN: Asian; Ad: Admixed

**Supplementary Table 4:**

| **#VUS** | | | | | | | | | |
| --- | --- | --- | --- | --- | --- | --- | --- | --- | --- |
|  | **#VUS_a** | | | **#VUS_b** | | | **#VUS_c** | | |
| *Predictors* | *Incidence Rate Ratios* | *CI* | *p* | *Incidence Rate Ratios* | *CI* | *p* | *Incidence Rate Ratios* | *CI* | *p* |
| (Intercept) | 1.00 | 0.78 – 1.28 | 0.981 | 1.39 ^*^ | 1.00 – 1.89 | **0.041** | 2.34^***^ | 1.99 – 2.74 | **<0.001** |
| Asian | 1.61 ^*^ | 1.09 – 2.32 | **0.013** | 1.81 ^*^ | 1.05 – 2.93 | **0.022** |  |  |  |
| America | 4.58 ^*^ | 1.30 – 13.76 | **0.011** |  |  |  |  |  |  |
| Family history of non-breast / ovarian cancer  [yes] | 1.35 ^*^ | 1.06 – 1.75 | **0.019** | 1.60 ^**^ | 1.15 – 2.29 | **0.007** |  |  |  |
| Observations | 276 | | | 98 | | | 65 | | |
| R^2^ Nagelkerke | 0.077 | | | 0.261 | | | -0.000 | | |
| ** p<0.05   ** p<0.01   *** p<0.001* | | | | | | | | | |

1. **#VUS_a** - Model summary when all patients are selected b) **#VUS_b -** selected patients with VUS in genes that form top 10% in the cohort (c) **#VUS_c -** patients with VUS in the 10 (is not 9?) genes associated to risk of breast cancer (33471991).

**Supplementary Table 5:**

| **logistic regression, pathogenic** | | | | | | |
| --- | --- | --- | --- | --- | --- | --- |
|  | **P\LP - full model test** | | | **P\LP - final model** | | |
| *Predictors* | *Odds Ratios* | *CI* | *p* | *Odds Ratios* | *CI* | *p* |
| (Intercept) | Inf | 0.00 – Inf | 0.678 | 0.33 ^***^ | 0.25 – 0.42 | **<0.001** |
| European | 0.00 | 0.00 – Inf | 0.678 |  |  |  |
| African | 0.00 | 0.00 – Inf | 0.678 |  |  |  |
| Asian | 0.00 | 0.00 – Inf | 0.678 |  |  |  |
| America | 0.00 | 0.00 – Inf | 0.678 |  |  |  |
| Ashkenazi Jewish ethnicity [yes] | 10.88 ^*^ | 1.31 – 228.43 | **0.044** | 9.19 | 1.16 – 187.30 | 0.056 |
| Age at diagnosis years | 0.98 | 0.96 – 1.01 | 0.224 |  |  |  |
| Family history of breast / ovarian cancer [yes] | 1.45 | 0.77 – 2.79 | 0.260 |  |  |  |
| Family history of non-breast / ovarian cancer [yes] | 0.99 | 0.52 – 1.95 | 0.965 |  |  |  |
| Observations | 274 | | | 321 | | |
| R^2^ Tjur | 0.029 | | | 0.017 | | |
| AIC | 319.836 | | | 362.246 | | |
| ** p<0.05   ** p<0.01   *** p<0.001* | | | | | | |
